# A Study of the Talent Training Project Management for Semiconductor Industry in Taiwan: The Application of a Hybrid Data Envelopment Analysis Approach

**DOI:** 10.1155/2014/296345

**Published:** 2014-04-08

**Authors:** Ling-Jing Kao, Shu-Yu Chiu, Hsien-Tang Ko

**Affiliations:** ^1^Department of Business Management, National Taipei University of Technology, Taipei 10608, Taiwan; ^2^Department of Business Administration, Fu Jen Catholic University, Taipei 24205, Taiwan; ^3^Industry Support Division, Institute for Information Industry, Taipei 11503, Taiwan

## Abstract

The purpose of this study is to evaluate the training institution performance and to improve the management of the Manpower Training Project (MTP) administered by the Semiconductor Institute in Taiwan. Much literature assesses the efficiency of an internal training program initiated by a firm, but only little literature studies the efficiency of an external training program led by government. In the study, a hybrid solution of ICA-DEA and ICA-MPI is developed for measuring the efficiency and the productivity growth of each training institution over the period. The technical efficiency change, the technological change, pure technical efficiency change, scale efficiency change, and the total factor productivity change were evaluated according to five inputs and two outputs. According to the results of the study, the training institutions can be classified by their efficiency successfully and the guidelines for the optimal level of input resources can be obtained for each inefficient training institution. The Semiconductor Institute in Taiwan can allocate budget more appropriately and establish withdrawal mechanisms for inefficient training institutions.

## 1. Introduction 

Due to the fast growth and expansion of high-tech industry in Taiwan, high-tech companies in the semiconductor industry have a strong demand for technical and R&D talents, but the academic institutions, the major supplier of high-tech manpower, cannot train enough students to fulfill this demand. According to an investigation conducted by Taiwan's Science and Technology Advisory Group of Executive Yuan in 2001, the technical manpower shortage for the semiconductor industry alone was about 6,600 people during 2003 to 2005. Facing severe global competition, both Taiwan authorities and the industry must find another way to cultivate more high-tech talents efficiently to pursue sustainable development and to maintain competitive advantage. Therefore, the Industrial Development Bureau (IDB) at the Ministry of Economic Affairs established the Semiconductor Institute to implement the Manpower Training Project (MTP) for matching up with the “Challenge 2008 National Development Plan” of Executive Yuan in 2003.

The main objective of the MTP is to fulfill the shortage of semiconductor manpower by providing training classes to those who want to pursue a career in the semiconductor industry. The career training program was carried out by various training institutions, which are affiliated with university/college, research institutions, or grassroots organizations, in northern, central, and southern Taiwan. During the year of 2003 to 2005, MTP's career training program has teamed up with over 20 training institutions to provide 283 classes with different training lengths. 3,950 graduates of the MTP have made contributions to the semiconductor industry.

Even though the MTP has been implemented for years and the success of the MTP has been evidenced by the accumulated number of trainees since 2003, the Semiconductor Institute encounters several challenges in project management. First, no scientific evaluation method has been established for measuring the project implementation efficiency of training institutions which are responsible for course design, student enrollment, and student job replacement. Second, no official withdrawal mechanism for inefficient training institutions and an optimal resource allocation are developed for improving the overall performance of the Manpower Training Project (MTP).

The issues faced by the Semiconductor Institute are not unusual for project management in general. Like the MPT, a project could be administered by a project manager in one organization (like the Semiconductor Institute), but the implementation of this project is assigned to multiple decision-making units (like the training institutes) with the same objective, but different competence and execution ability. Decision-making units receive the guidance and financial support from the project manager, and they will be evaluated by the project manager at the end of the project execution period as well. The example includes the project management in the health care and in the financial service industry.

To evaluate the performance of decision-making units, various efficiency measurement tools, such as conventional statistical methods, nonparametric methods, and artificial intelligence methods, have been successfully developed in the literature. Among these tools, the data envelopment analysis (DEA) approach has received the most discussion. DEA is known as the efficient frontier approach [1, 2]. The term “envelopment” refers to the idea that inefficient DMUs (decision-making units) are located inside an area enveloped by the efficient DMUs. DEA is constructed based on the concept of relative efficiency, which is defined as the ratio of the weighted sum of outputs to the weighted sum of inputs [2].

Even though DEA has been applied in efficiency measurement successfully, the presence of strong correlation among the input variables of a DMU can bias the efficiency estimates of a DMU in the slack analysis [3, 4]. To alleviate this problem, a two-stage integrated approach of independent component analysis (ICA) and data envelopment analysis (DEA) has been proposed by Kao et al. [5] in the literature. In their work, a simulated dataset and an empirical hospital dataset were used to demonstrate the validity of the integrated ICA and DEA approach. Their results show that the integrated ICA and DEA method can not only separate performance differences between the DMUs but also outperform principal component analysis (PCA-) variable reduction in its discrimination performance.

In this study, to evaluate the training institution performance administered by the Semiconductor Institute in Taiwan, we first applied the PCA-ICA technique proposed by Kao et al. [5]. Then, we extended their technique by using Malmquist productivity indices (MPI) to measure the productivity growth of each DMU during multiple times. In other words, in this paper, we propose a hybrid solution of ICA-DEA and ICA-MPI to address the project management issues for the Semiconductor Institute. The proposed solution consists of three steps. First, we used ICA to convert the input data of the training institutions in 2009 into separate independent signals, called independent components (ICs). Then, these independent components enter the DEA approach as input variables to measure efficiency. The outcome of DEA, the slack analysis, is used to determine the optimal levels of input resources for each training institution and having training institutions adjust their resource allocation accordingly. Finally, MPI is used to determine whether the performance improvement of a training institution is caused by the suggestion of the slack analysis.

The proposed solution is illustrated by a dataset provided by the Semiconductor Institute in Taiwan. The dataset contains the input and output information of ten training institutions which joined the Semiconductor Institute's Manpower Training Project in 2009 and 2010. Five input variables required to deliver the training course include the total number of professional-qualified faculty, the total number of academic-qualified faculty, the total number of administrative staffs, the average practical training hours, and the total number of graduates who majored in semiconductor-unrelated fields. Two project outputs are the total number of successful employment placements and the total number of graduates from the training institution.

In the empirical study, we compared the outcomes of ICA-DEA and single DEA to demonstrate the influence of correlated input variables on the discrimination capability of DEA. Our analysis shows that ICA-DEA approach can avoid efficiency misjudgment. They are consistent with the conclusion made by Kao et al. [5]. (The ICA solution extracts the original source signal to a much greater extent than the PCA solution.) The analysis of ICA-MPI also indicates that the productivity of the most inefficient training institutions is apparently enhanced in 2010 if the suggestions of resource allocation from ICA-DEA were taken. According to our analysis, the Semiconductor Institute could consider reallocating budget by each training institution's efficiency in project execution and to withdraw those inefficient training institutions which show no improvement.

This paper contributes to the literature and the semiconductor industry in two aspects. First, the proposed hybrid approach of ICA-DEA and ICA-MPI can be applied to study the efficiency of DMUs and help the project manager to manage his project more appropriately (e.g., provide improvement suggestion). The proposed approach can also be extended to other similar applications in business. For example, a firm's HR department can use the proposed approach to evaluate the efficiency of a HR training project in multiple campuses. Second, the proposed approach can provide guidance of resource allocation in project management. For example, the Semiconductor Institute can set up optimal input levels for each training institution to maximize the number of job replacements and the number of graduates. The project management practice of the Semiconductor Institute can also be extended to other government projects.

The rest of this paper is organized as follows: Section 2 gives an introduction to project performance evaluation.  Section 3 gives a brief introduction to data envelopment analysis (DEA), Malmquist productivity indices (MPI), and independent component analysis (ICA). In Section 4, we begin by developing the proposed hybrid model and comparing it to single DEA with the training institution data provided by the Semiconductor Institute in Taiwan. Managerial implications and conclusions are offered in Section 5.

## 2. Project Performance Evaluation

Most project management literature focus on project management strategies, project planning and control, process improvement, risk management, simulation modeling, leadership and team building, or negotiation and contracting strategy. Due to the temporary nature of a project, not much literature is concerned about project efficiency evaluation and performance enhancement for some projects, like the Manpower Training Project in Taiwan, which is temporary for the project execution organization (training institutions) but is recursive for the organization (the Semiconductor Institute) which administers this project.

However, regardless of industry application, evaluating project performance is critical to any organization because project managers can avoid making similar mistakes from experience. Then if projects with the same properties will be executed again, the entire process of project management can be done much smoothly and efficiently.

In the literature, data envelopment analysis (DEA) has been widely used as a benchmarking approach in evaluating project productivity performance [6]. For example, Swink et al. [7] employed a sequential data envelopment analysis (DEA) methodology that simultaneously incorporates multiple factors to study efficiency and performance tradeoffs for new product development projects. Paradi et al. [8] applied DEA to measure the efficiency of software production at two large Canadian banks. Banker et al. [9] and Herrero and Salmeron [10] used DEA to analyze software project efficiency. Cao and Hoffman [11] applied DEA in a project performance evaluation system for Honeywell Federal Manufacturing & Technologies. Vitner et al. [12] implemented DEA within the multidimensional control system (MDCS) and the earned value management system (EVMS) to evaluate the performances of projects in a multiproject environment, where each project is usually a one-time nonrepeated event and has its own inputs and outputs. El-Mashaleh et al. [13] utilized DEA to evaluate safety performance of construction contractors. Ghapanchi et al. [14] applied DEA on the static portfolio selection problem in project management. Sowlati et al. [15] presented a new solution within the data envelopment analysis framework for prioritizing information system projects.

Along with DEA's popularity, DEA's two drawbacks must be acknowledged. First, when the strong correlation among inputs of a DMU is observed, the result of slack analysis can be biased [3, 4]. It is because DEA used the weighting method to calculate the ratio between inputs and outputs of each DMU. Second, DEA's discrimination capability is lessened if the model is misspecified or the number of DMUs is too small.

Besides, these applications in the literature are not suitable for managing a project which has the following characteristics. First, the project itself is administered and executed with minor adjustment recursively over a period of time. Second, the project is planned, organized, led, and controlled by one administrative organization but is executed by multiple execution institutions which are not affiliated with the administrative organization. Third, the administrative organization allocates budget to execution institutions periodically, evaluates the performance of each execution institution, and decides if the contract with execution institutions should be renewed. Fourth, the administrative organization can provide improvement suggestion to inefficient execution institutions, but inefficient execution institutions may not necessarily follow the improve suggestions due to some unobservable factors.

Therefore, a new method of project performance evaluation is needed. In this research, we propose a hybrid method which combines ICA, DEA, and MPI together to solve the project performance evaluation problems as illustrated above. In the following section, a brief introduction of each method is provided.

## 3. Methodology

### 3.1. DEA

Data envelopment analysis is known as the efficient frontier approach [1, 2]. The term “envelopment” refers to the idea that inefficient DMUs (Decision Making Units) are located inside an area enveloped by the efficient DMUs. DEA is constructed based on the concept of relative efficiency, which is defined as the ratio of the weighted sum of outputs to the weighted sum of inputs [2]. The solution of DEA requires that the weights for inputs and outputs of each unit are selected to maximize its efficiency under certain constraints. Thus, the mathematical programming form of the BCC primal input-oriented model is formulated as follows [1, 16, 17]:

(1) Minimize  θSubject  to  ∑j=1nXjλj≤θX0,  ∑j=1nYjλj≥Y0,  ∑j=1nλj=1,  λj≥0,

where *X*_*j*_ = (*x*_1*j*_, *x*_2*j*_,…, *x*_*mj*_) and *Y*_*j*_ = (*y*_1*j*_, *y*_2*j*_,…, *y*_*rj*_) represent the observed inputs and outputs of production units *j* = 1,…, *n*. In this primal model the efficiency score *θ* of production unit (*X*_0_, *Y*_0_) is found; (*X*_0_, *Y*_0_) is any unit from the set of production units (*X*_*j*_, *Y*_*j*_), *j* = 1,…, *n*.

DEA and its modification have been increasingly used over the past decade to measure performance. For example, DEA has been applied to evaluate the performance of supply chain [18], chains stores [19], e-commerce [20], and coal-fired power plants [21]. Besides, Avkiran and Rowlands [22] used DEA and stochastic frontier analysis to investigate how organizational performance varies with the operating environment, statistical noise, and managerial efficiency.

Along with DEA's popularity, DEA's two drawbacks must be acknowledged before it is applied. First, when the strong correlation among inputs of a DMU is observed, the result of slack analysis can be biased. It is because DEA used the weighting method to calculate the ratio between inputs and outputs of each DMU. Second, DEA's discrimination capability is lessened if the model is misspecified or the number of DMUs is too small.

Even though Adler and Golany [3, 4] have suggested using the principal component analysis (PCA) to produce uncorrelated linear combinations of original inputs, PCA only considers second order moments but lacks information on higher order statistics [23]. Thus, in this research, we adopt the ICA solution proposed by Kao et al. [5] to solve the input correlation problem.

### 3.2. Malmquist Productivity Index

Productivity is a relative concept which is used to measure, analyze, and monitor a DMU's project execution ability relative to itself in the past year or to other DMUs at the same year. Even though productivity can be defined in various ways, the Malmquist productivity index (MPI), which was introduced by Malmquist [24] and was further integrated into the nonparametric framework by Caves et al. [25], Färe et al. [26], Färe et al. [27], and Cooper et al. [1], has become the standard approach in the literature lately.

Based on multi input-output frontier representations of the production technology [28], the Malmquist productivity index has many advantages. First, MPI allows us to measure productivity progress or regress over time and compare productivities among multiple DMUs. Second, MPI can be decomposed into two components: technical efficiency change and technical change, which provides researchers with some information on the causes of productivity change.

According to Färe et al. [27], MPI is defined by the distance function *D* and is expressed as follows:

(2) Mo(xt+1,yt+1,xt,yt) =Dot+1(xt+1,yt+1)Dot(xt,yt)  ×[Dot(xt+1,yt+1)Dot+1(xt+1,yt+1)Dot(xt,yt)Dot+1(xt,yt)]1/2,

where *x* ^*t*^ ∈ *R*_+_^*n*^ and *y* ^*t*^ ∈ *R*_+_^*m*^ denote the input vector and the output vector of a DMU at time *t*, respectively. The term outside the brackets, called “Efficiency Change (Eff-Ch),” is the ratio of two distance functions which measures change in the technical efficiency between time *t* and time *t* + 1. The term within the brackets, called “Technology Change (Tech-Ch),” is a measure of the technical change in the production technology between time *t* and time *t* + 1. MPI can be interpreted as a measure of total factor productivity (TFP) growth. For both Eff-Ch and Tech-Ch, a value greater than, equal to, or less than one indicates improvement, no change, and deterioration in performance over time.

Färe et al. [27] redefined “Efficiency Change (Eff-Ch)” as follows:

(3) Dot+1(xt+1,yt+1)Dot(xt,yt) =[Dot+1(xt+1,yt+1)Dot(xt,yt)]  ×{[Doct+1(xt+1,yt+1)/Dot+1(xt+1,yt+1)][Doct(xt,yt)/Dot(xt,yt)]}.

As seen in (3), four difference distance functions are included in the calculation of MPI for two adjacent periods *t* and *t* + 1. To define the distance functions, let us assume that the set of production possibilities of a DMU at time *t* is defined as follows:

(4) $$\begin{array}{c} S^{t}=\left\{\left(x^{t},y^{t}\right)\;|\;x^{t}\;\;\text{can}\;\;\text{produce}\;\;y^{t}\right\}. \end{array}$$

And Shephard [29] or Färe et al. [26] define the output distance function at time *t* as follows:

(5) Dot(xt,yt)=inf⁡⁡{θ ∣ (xt,ytθ)∈St}=(sup⁡{θ ∣ (xt,θyt)∈St})−1.

Note that, in (5), *D*_*o*_^*t*^(*x* ^*t*^, *y* ^*t*^) ≤ 1 if and only if (*x* ^*t*^, *y* ^*t*^) ∈ *S* ^*t*^, and *D*_*o*_^*t*^(*x* ^*t*^, *y* ^*t*^) = 1 if and only if (*x* ^*t*^, *y* ^*t*^) is on the frontier of the technology, which suggests that a DMU is technically efficient.

For “Technology Change (Tech-Ch)” in (2), distance functions with respect to two different time periods are defined as follows:

(6) $$\begin{array}{c} D_{o}^{t}\left(x^{t+1},y^{t+1}\right)=\inf\left\{\theta \;|\;\left(x^{t+1},\frac{y^{t+1}}{\theta }\right)\in S^{t}\right\}, \end{array}$$

(7) $$\begin{array}{c} D_{o}^{t+1}\left(x^{t},y^{t}\right)=\inf\left\{\theta \;|\;\left(x^{t},\frac{y^{t}}{\theta }\right)\in S^{t+1}\right\}. \end{array}$$

Equation (6) is the distance function used to measure the maximal proportional change in output given that (*x* ^*t*+1^, *y* ^*t*+1^) is feasible in relation to technology at time *t*. And (7) is the distance function which is used to measure the maximal proportional change in output requirement given that (*x* ^*t*^, *y* ^*t*^) is feasible in relation to technology at time *t* + 1.

Among various methods used to measure the distance functions, which make up MPI, the DEA-like method [30, 31] which only relies on minimum assumptions regarding the shape of the production frontier is most widely adopted. In this paper, we followed this convention and used mathematical programming software DEAP to obtain the results of MPI for the empirical study.

### 3.3. ICA

ICA can be viewed as an extension of principal component analysis (PCA) with a different objective [32]. PCA is a dimension reduction technique that reduces the data dimension by projecting the correlated variables into a smaller set of new variables that are uncorrelated and retain most of the original variance. Thus PCA can only decorrelate variables, not making principle components independent. ICA is essentially a novel statistical signal processing technique used to extract independent sources from observed multivariate statistical data where no relevant data mixture mechanisms are available [32, 33].

ICA is a methodology for capturing both second and higher order statistics, and it projects the input data onto the basis vectors that are as statistically independent as possible [34, 35]. These characteristics of ICA distinguish ICA from PCA which is used to find a set of the most representative projection vectors such that the projected samples retain the most information about the original samples [36].

The literature has applied ICA in human face recognition on FERET database [34, 37] and the Olivetti and Yale databases [38]. In the latter study, Liu and Wechsler [37] and Bartlett et al. [34] have shown that ICA outperforms PCA even though Moghaddam [39] states that the performances of ICA and PCA have no significant difference.

For illustrative purpose, we can assume that each of *m* measured variables is given as a linear combination of *n* (≤*m*) unknown independent components. The independent components and the measured variables are zero mean. The relationship between a measured-variable data matrix *X* and an independent-component data matrix *S* is given by *X* = *AS*, where *A* is an unknown full-rank matrix, called the mixing matrix. The ICA model aims at finding a demixing matrix *W* such that *Y* = *WX*. The row vectors in *Y* must be as statistically independent as possible and are called independent components (ICs). The ICs are used to estimate the latent variables *s*_*i*_ (the *i*th row vector in matrix *S*). Basically, the ICA modeling is formulated as an optimization problem by setting up the measure of statistical independence of ICs as an objective function and using some optimization techniques to solve the demixing matrix *W* [40, 41].

Typically, the statistical independence of ICs can be measured in terms of their non-Gaussian properties [32, 33] and the non-Gaussianity can be verified by two common statistics: kurtosis and negentropy. In this study, a fixed-point algorithm [33] which maximizes the kurtosis is used to estimate the separating matrix *W*. For more detailed information about the fixed-point algorithm, please refer to Hyvärinen et al. [33].

## 4. Research Methodology and Empirical Application

**Figure 1:**
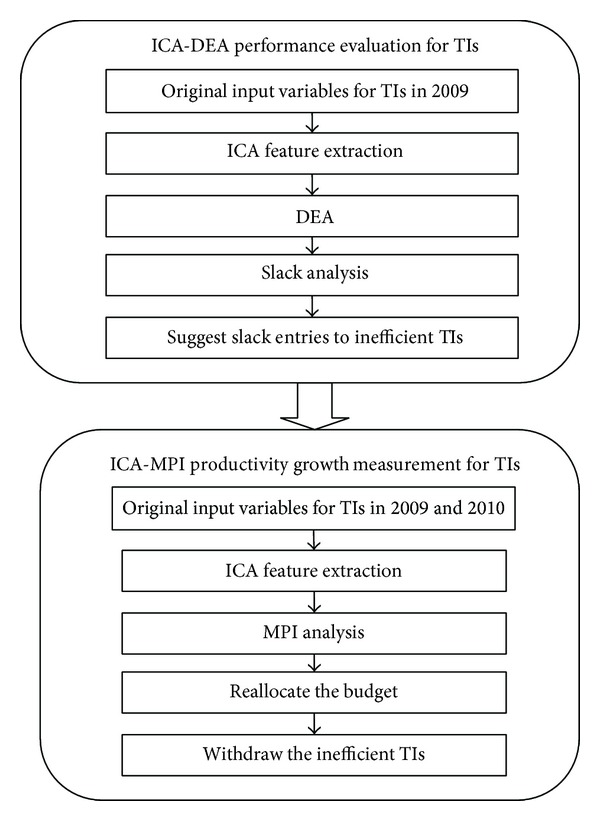
The research scheme of the proposed analysis model.

The schematic representation of the proposed model is illustrated in Figure 1. As shown in the figure, there are two stages in our proposed approach. The first stage is suggested by Kao et al. [5]. We used ICA to convert observed input data of the training institutions in 2009 into separate independent signals, called independent components (ICs). Then, these independent components enter the DEA approach as input variables to measure efficiency. Finally the slack entries (optimal levels of input resources) derived from the DEA approach can be used to suggest the areas required improvement to inefficient TIs.

In the second stage, ICA is applied to the input variables of TIs in 2009 and 2010 for generating ICs simultaneously. The estimated ICs, regarded as the key factors affecting productivity growth, are then utilized as new input variables in the Malmquist productivity index (MPI) to see if the productivity of inefficient TIs in 2009 could apparently grow in the following year (2010) after the suggested slack entries are considered. Finally, Semiconductor Institute could consider reallocating budget for each TI based on its corresponding efficiency and withdrawing those inefficient TIs that fail to improve in 2010.

### 4.1. Data

In this study, the dataset of training institutions in 2009 and 2010 provided by the Semiconductor Institute in Taiwan is used to illustrate the proposed ICA-DEA and ICA-MPI approaches. The data contains the information of ten training institutions which joined the Semiconductor Institute's Manpower Training Project in 2009 and 2010. According to their functional complexity, these ten training institutions can be categorized into three different classes: (1) universities and colleges; (2) research institutions; and (3) grassroots organizations. Among these ten training institutions, six are located in northern Taiwan while four are located in southern Taiwan.

Because, regardless of methodologies, the result of efficiency measurement is highly influenced by the selection of input and output variables, reviewing the literature for variable selection in similar studies is needed. We found that, in the literature, the DEA has been applied in studying school efficiency [42–44] which is the closest to our study. Thus, we followed the suggestion in the literature of school efficiency and chose five input and two output variables to calculate the efficiency of each training institution. The variables adopted in this study are defined and explained in Table 1. For confidentiality reason, the datasets have been linearly rescaled. The rescaled results are given in Table 2.

Practically, subsets of the inputs or outputs are always correlated. The high degree of correlations between the variables could cause issues with the distribution of the weights. Dropping a highly correlated variable from the assessment could not only reduce the efficiency ratings for some DMUs [45] but also lead to significant changes in efficiencies [46]. Therefore, examining the correlation among variables becomes necessary before the further analysis is preceded.

The correlation matrix of input and output variables in our study is provided in Table 3. It shows that there are positive correlations among all of the variables. The lowest significant correlation coefficient is 0.1809 which is between Industry_faculty (**x**_1_) and Academic_faculty (**x**_2_). The highest significant coefficient is 0.9475 which is between the successful employment (**y**_1_) and Trainee_# (**y**_2_). Therefore, there is a need to reduce the degree of correlation before conducting DEA.

### 4.2. Efficiency Computations and Slack Analysis for the Training Institution in 2009

We used the BCC input-oriented model in the* DEA-PRO* software, which is proposed by Banker et al. [16], to compute efficiency scores. To demonstrate the validity of the proposed model, the performance of the proposed ICA-DEA method is compared to the single DEA model in this section. The single DEA model simply applies the DEA model to measure the efficiency of the training institutions, without using ICA as a preprocessing tool.

For the proposed ICA-DEA model, we first applied the basic ICA approach to estimate a de-mixing matrix** W** and five independent components (${\tilde{b}}_{1}$, ${\tilde{b}}_{2}$,…, and ${\tilde{b}}_{5}$). In order to select more meaningful ICs, the statistical independence of ICs is evaluated by computing the kurtosis values of the ICs herein. The estimated five kurtosis values for the ICs are 5.618, 5.217, 4.3014, 2.2178, and 2.0338. The larger an IC's kurtosis value is, the more important the IC is [47]. Thus, IC_1_, IC_2_, and IC_3_ (i.e., ${\tilde{b}}_{1}$, ${\tilde{b}}_{2}$, and ${\tilde{b}}_{3}$), regarded as key factors affecting the results of efficiency measurement, are used as three new input variables for the DEA model. Note that the extracted IC might have negative values which violate the semipositive assumption for the DEA model; that is, all inputs and all outputs are nonnegative, and at least one input and one output are positive. To solve this problem, we simply subtract each IC_*i*_ from its corresponding minimum value, that is, min(IC_*i*_).

The results of efficiency measurement for both single DEA method and ICA-DEA method are reported in Table 4. It shows that, compared to the single DEA method which identified 7 efficient TIs, the ICA-DEA method only identified 5 efficient TIs. In addition, the single DEA method produced a higher average efficiency score and smaller standard deviation than the ICA-DEA method did. These results indicate that the single DEA method fails to discriminate the performance difference among training institutions. After considering the correlation among variables, ICA-DEA method can truly enhance DEA method's ability to identify inefficient TIs.

DEA provides not only the efficiency results but also slack analysis, by which guidelines for the optimal level of input and output resources can be derived for each training institution. That is, each training institution could have its input and output resources set at the optimal level—the original level minus the inefficient and slack amounts from the DEA results [48].

Table 5 reports the final results of slack analysis for all ten training institutions by using the ICA-DEA approach. It is noticed that, because of the adoption of the ICA technique, the slack entries generated by the ICA-DEA model need to be retransformed in order to obtain the optimal level of input resource for improving efficiency scores. In the retransformed procedure, the slack entries generated by the ICA-DEA model are defined as Δ**S**_*i*_. Then, based on the relationship between input resources and independent component, *X* = *SA*, we formulate our retransforming procedure as Δ*X*_*i*_ = Δ*S*_*i*_ *A*.

Table 5 shows that all inefficient TIs have negative slack entries, which suggests that all inefficient TIs need to reduce their input levels in Industry_faculty (**x**_1_), Academic_faculty (**x**_2_), Administrative_staffs (**x**_3_), Project_hours (**x**_4_), and Student_# in unrelated field (**x**_5_). This result is reasonable for the following reasons. First, compared to those with semiconductor-related major, students with unrelated major have weaker knowledge foundation. Given the same training time, students without related background are harder to reach the same training outcome. Therefore, the performance of an inefficient TI can be enhanced if it can recruit more students with semiconductor-related major. Second, some course projects are redundant to students who have taken similar projects in college. Therefore, TI's performance can be enhanced if they can remove the redundancy and only keep training courses complement with each other. The suggestion of slack analysis was later delivered to those inefficient training institutions by Semiconductor Institute for seeking their significant improvement.

### 4.3. Measuring Productivity Growth in Training Institutions

**Figure 2:**
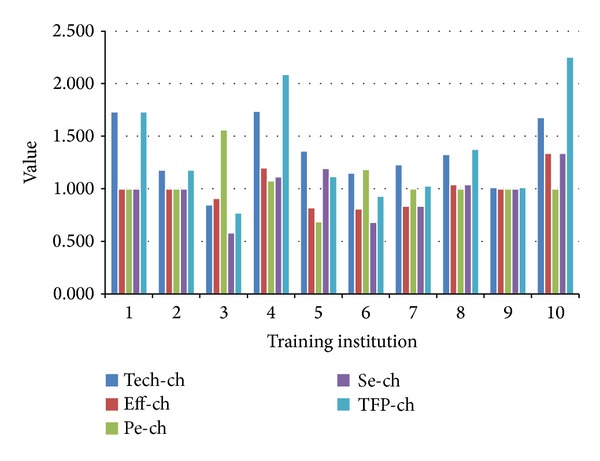
Malmquist index summary of studied training institution.

The Malmquist productivity indices explained in Section 3 are applied to estimate the TFP growth rates for the training institutions in the period 2009-2010. The Malmquist total factor productivity (TFP) change indexes are calculated using DEAP 2.1 linear program developed by Coelli et al. [31]. The Malmquist indices of all ten institutions are presented in Table 6 and Figure 2. The table contains the TFP changes and its components for the training institutions for the entire time period. As mentioned before, the index value of TFP less than 1 indicates performance deterioration in 2010 while the index value of TFP greater than 1 indicates performance improvement in 2010.

As shown in Table 6, the average “TFP-ch” increased by 27.1% which indicates that, on average, the overall “TFP-ch” of TIs has improved for the entire period of 2009-2010. This result indicates that the slack entries suggested by Semiconductor Institute to those inefficient training institutions do improve their performance.

Table 6 also shows that all TIs, except the 3rd TI, have great advance in technological change, and it leads to the average “Tech-ch” of 29.3%. Among all TIs, the greatest increase in the “Tech-ch” values during the period from 2009 to 2010 was the 4th training institution. The reason of such an increase is the low technical efficiency in 2009 with a value of 0.5213. According to the “Tech-ch” values, the least increase changes were seen for the 9th and the 6th training institutions. The only TI ruled towards a decrease (with a rate of 15.4%) is the 3rd training institution.

When the values of “Eff-ch” are examined, the 10th training institution had the largest increase in “Eff-ch” with a rate of 34.0% while the other training institutions were almost detected with a decrease. The “Eff-ch” values of the 6th, 5th, 7th, and 3rd training institution were decreased by 19.1%, 17.9%, 16.5%, and 9.1%, respectively. For “Pe-ch,” the 3rd, 6th, and 4th training institutions show an increase of 56.2%, 18.5%, and 7.7%, respectively, while the 5th training institution had a decrease of 31.3%. There is no change seen in the “Pe-ch” values of other training institutions during the same period of time.

For the values of “Se-ch,” the 10th training institution had the largest increase with a rate of 34.0%. The “Se-ch” values of the 5th, 4th, and 8th training institutions were increased by 19.5%, 11.4%, and 4.0%, respectively. This situation reveals that the training institution had gained success in means of production realized via appropriate scale adjustment.

Table 6 also provides the “Tfp-ch” values of all training institutions. According to Table 6, the average value of “Tfp-ch” revealed an increase of 27.1%. In particular the 10th training institution has the greatest increase of 125.3% in “Tfp-ch.” Among ten training institutions, only the 3rd and 6th training institutions had shown a decrease of 23.1% and 6.9%, respectively.

In summary, the following observations can be reached by MPI.

1. The 4th and 10th training institutions have the highest values of “Tech-ch” while the 3rd training institution has the lowest value of “Tech-ch.” Thus, it is concluded that the 4th and 10th training institutions had gained success in catching up the production limits.
2. The 10th training institution has the highest “Eff-ch” value, and the 6th training institution has the lowest “Eff-ch” value.
3. The 3rd training institution has the highest “Pe-ch” value while the 5th training institution has the lowest “Pe-ch” value.
4. The 10th training institution has the highest “Se-ch” value while the 3rd training institution has the lowest “Se-ch” value.
5. The 10th training institution has the highest “Tfp-ch” value while the 3rd training institution has the lowest “Tfp-ch” value.

From our analysis, we can conclude that the most successful training institution is the 10th training institution due to its improvement in “Eff-ch,” “Se-ch,” and “Tfp-ch.” And, the 3rd and 4th training institutions are the most successful training institution due to their improvement in “Pe-ch” and “Tech-ch,” respectively. Among these ten training institutions, the 10th training institution is worthy of gaining more budget from Semiconductor Institute because it takes the suggested slack entries from analysis and becomes the most successful training institution. Moreover, the 3rd and 6th training institutions were considered to be withdrawal because they do not meet expected targets during execution.

## 5. Managerial Implications and Conclusions

### 5.1. Managerial Implications

While much can be accomplished at the project management perspective, several policy implications and conclusions can emerge from this study. All of these recommendations must be considered in light of the context and goals of the project in which they are applied. Thus, the following suggestions are offered.

First, our research suggests that the proposed ICA-DEA method can provide a robust assessment of project performance because the ICA-DEA method has more ability to distinguish between the performances of training institutions and to help the project manager to manage and evaluate his project more appropriately.

Second, the slack analysis of ICA-DEA provides a clear guidance for resource allocation. Our application evidences that the slack analysis of ICA-DEA can enhance the overall productivity of the Manpower Training Project. The Semiconductor Institute in Taiwan can apply the proposed method to evaluate training institutions during their execution period.

Finally, ICA-DEA and ICA-MPI can help a project manager establish a scientific withdrawal mechanism for inefficient DMUs. In our study, Manpower Training Project (MTP) can be executed more effectively and the resources can be allocated more reasonably.

### 5.2. Conclusions

In this research, a hybrid solution of ICA-DEA and ICA-MPI is proposed to evaluate productivity of a project which has the following characteristics. First, the project itself is administered and executed with minor adjustment recursively over a period of time. Second, the project is planned, organized, led, and controlled by one administrative organization but is executed by multiple execution institutions which are not affiliated with the administrative organization. Third, the administrative organization allocates budget to execution institutions periodically, evaluates the performance of each execution institution, and decides if the contract with execution institutions should be renewed. Fourth, the administrative organization can provide improvement suggestion to inefficient execution institutions, but inefficient execution institutions may not necessarily follow its improvement suggestion due to some unobservable factors. The example of this type project includes the project performance evaluation in the health care and in the financial service industry.

In summary, this research contributes to the project management literature in three aspects. First, the proposed hybrid approach of ICA-DEA and ICA-MPI can be applied to study the efficiency of DMUs and help the project manager to manage his project more appropriately (e.g., provide improvement suggestion). Second, the proposed approach can be extended to other similar applications in business. For example, a firm's HR department can use the proposed approach to evaluate the efficiency of a HR training project in multiple campuses, or a firm can use the proposed approach to evaluate outsourcing performance of various business partners. Finally, the proposed approach can provide guidance of resource allocation in project management.

In the future research, our methodology can be further developed in the following direction. First, due to the data limitation, the empirical study can only demonstrate the proposed hybrid approach in two time periods. The model's performance assessment for multiple time periods can be considered in the future study. Second, our methodology can be extended to evaluate the qualification of a potential contractor (e.g., training institutions) to implement the project. It can reduce the risk of the Semiconductor Institute. Finally, DEA is a nonparametric approach which cannot deal with the stochastic changes which may affect project efficiency and resource allocation over time. Therefore, the stochastic techniques are suggested to be incorporated into the model development.

## Figures and Tables

**Table 1:** Definition and explanation of variables.

	Variables	Definition and explanation
Inputs	Industry_faculty (**x**_1_)	The total number of professionally qualified faculty from the industry within a training institution
	Academic_faculty (**x**_2_)	The total number of academically qualified faculty from universities within a training institution
	Administrative_staffs (**x**_3_)	The total number of administrative staffs employed in a training institution
	Project_hours (**x**_4_)	The average hours of the course project which each student spent in practical training
	Student_# in unrelated field (**x**_5_)	The total number of graduates who majored in semiconductor-unrelated fields

Outputs	Successful employment (**y**_1_)	The total number of successful employment placements
	Trainee_# (**y**_2_)	The total number of trainees graduated from a training institution

**Table 2:** Rescaled input and output variables and their summary statistics.

Year	Training institution	**x**_1_	**x**_2_	**x**_3_	**x**_4_	**x**_5_	**y**_1_	**y**_2_
2009	1	0.2300	0.0496	0.4720	0.0100	0.1600	0.0100	0.1623
	2	0.4500	0.5644	1.0000	0.5417	1.0000	0.8122	0.9086
	3	0.1200	0.2080	0.4940	0.2025	0.1000	0.0100	0.0252
	4	0.6700	0.3268	0.1640	0.8350	0.0700	0.1978	0.1775
	5	0.7800	0.3268	0.4500	0.6150	0.2200	0.3855	0.2689
	6	0.8900	0.0100	0.5380	0.6150	0.6700	0.1466	0.0709
	7	0.6700	0.1684	0.4940	0.5233	0.3700	1.0000	1.0000
	8	0.3400	0.4456	0.2080	0.3033	0.1300	0.7269	0.6040
	9	0.5600	0.2080	0.4280	0.5508	0.3400	0.3002	0.3298
	10	0.2300	0.5644	0.3620	0.3858	0.4000	0.2831	0.3451

2010	1	0.0100	0.2476	0.0100	0.1567	0.0700	0.1978	0.4669
	2	1.0000	1.0000	0.8900	1.0000	0.9400	0.7952	0.7715
	3	0.8900	0.6832	0.4940	0.8442	0.3100	0.0441	0.0100
	4	0.1200	0.1684	0.0760	0.1108	0.0100	0.1466	0.3755
	5	1.0000	0.1684	0.4060	1.1192	0.4000	0.5050	0.4822
	6	0.6700	0.1288	0.4720	0.5050	0.0700	0.1124	0.0862
	7	0.5600	0.3664	0.4060	0.6608	0.2500	0.9488	0.9238
	8	0.5600	0.2872	0.1420	0.3400	0.2800	0.3855	0.3908
	9	0.2300	0.0892	0.3400	0.3950	0.4300	0.9488	0.8020
	10	0.1200	0.6436	0.2960	0.1475	0.1900	0.3172	0.2842

Mean		0.5050	0.3327	0.4071	0.4931	0.3205	0.4137	0.4243
Std. Dev.		0.3162	0.2505	0.2396	0.3018	0.2738	0.3370	0.3144

**Table 3:** Correlation coefficients between variables.

	**x**_1_	**x**_2_	**x**_3_	**x**_4_	**x**_5_	**y**_1_	**y**_2_
**x**_1_	1	—	—	—	—	—	—
**x**_2_	0.1809	1	—	—	—	—	—
**x**_3_	0.4531	0.3849	1	—	—	—	—
**x**_4_	0.8936	0.3203	0.4205	1	—	—	—
**x**_5_	0.4464	0.4284	0.8159	0.4703	1	—	—
**y**_1_	0.2926	0.4754	0.4638	0.4215	0.6043	1	—
**y**_2_	0.3627	0.3995	0.5907	0.4463	0.7508	0.9475	1

**Table 4:** Summary of the results of single DEA and ICA-DEA models in 2009.

Year	Single DEA model	ICA-DEA model
Average score	0.9492	0.7514
Standard deviation	0.0859	0.2682
Maximum efficiency score	1	1
Minimum efficiency score	0.7778	0.3875
Number of efficient DMUs	7	5
Total number of DMUs	10	10
Percentage of efficient DMUs	70	50

**Table 5:** Slack analysis of ICA-DEA method for input variables.

Training institution	Industry_faculty (**x**_1_)	Academic_faculty (**x**_2_)	Admin_staffs (**x**_3_)	Project_hours (**x**_4_)	Student_# in unrelated field (**x**_5_)
1*	0	0	0	0	0
2	−1	−6	−20	−12	−15
3	0	−3	−5	−15	−3
4	−2	−3	−4	−35	−2
5*	0	0	0	0	0
6	0	−3	−3	−15	−5
7*	0	0	0	0	0
8*	0	0	0	0	0
9*	0	0	0	0	0
10	−6	−2	−6	−52	−7

*Efficient training institution.

**Table 6:** Malmquist indices of training institution.

Training institution	Technological change (Tech-ch)	Technical efficiency change (Eff-ch)	Pure technical efficiency change (Pe-ch)	Scale efficiency change (Se-ch)	Total factor productivity change (TFP-ch)
1*	1.732	1.000	1.000	1.000	1.732
2	1.178	1.000	1.000	1.000	1.178
3	0.846	0.909	1.562	0.582	0.769
4	1.740	1.200	1.077	1.114	2.088
5*	1.360	0.821	0.687	1.195	1.116
6	1.151	0.809	1.185	0.682	0.931
7*	1.231	0.835	1.000	0.835	1.028
8*	1.326	1.040	1.000	1.040	1.379
9*	1.014	1.000	1.000	1.000	1.014
10	1.681	1.340	1.000	1.340	2.253

Average	1.293	0.983	1.032	0.953	1.271

*Efficient training institution identified by ICA-DEA in 2009.
